# A single mutation in the 15S rRNA gene confers non sense suppressor activity and interacts with mRF1 the release factor in yeast mitochondria

**DOI:** 10.15698/mic2015.09.223

**Published:** 2015-08-02

**Authors:** Ali Gargouri, Catherine Macadré, Jaga Lazowska

**Affiliations:** 1 Centre de Génétique Moléculaire, Laboratoire propre du C.N.R.S associé à l'Université Pierre et Marie Curie. CNRS F-91198 Gif-sur-Yvette cedex, France.

**Keywords:** yeast, informational suppressors, 15S rRNA, nonsense, frame shift

## Abstract

We have determined the nucleotide sequence of the mim3-1 mitochondrial ribosomal suppressor, acting on ochre mitochondrial mutations and one frameshift mutation in *Saccharomyces cerevisiae*. The 15s rRNA suppressor gene contains a G633 to C transversion. Yeast mitochondrial G633 corresponds to G517 of the *E.coli *15S rRNA, which is occupied by an invariant G in all known small rRNA sequences. Interestingly, this mutation has occurred at the same position as the known MSU1 mitochondrial suppressor which changes G633 to A. The suppressor mutation lies in a highly conserved region of the rRNA, known in *E.coli *as the 530-loop, interacting with the S4, S5 and S12 ribosomal proteins. We also show an interesting interaction between the mitochondrial mim3-1 and the nuclear nam3-1 suppressors, both of which have the same action spectrum on mitochondrial mutations: nam3-1 abolishes the suppressor effect when present with mim3-1 in the same haploid cell. We discuss these results in the light of the nature of Nam3, identified by 1 as the yeast mitochondrial translation release factor. A hypothetical mechanism of suppression by "ribosome shifting" is also discussed in view of the nature of mutations suppressed and not suppressed.

## INTRODUCTION

Suppressors are divided into two wide groups: functional and informational. The former ones are located in a second gene, the product of which interacts functionally with the mutated product of the first gene. The latter ones act mainly during the translation step, resulting in the replacement of the mutated residue in the suppressed protein. Informational suppressors are frequently found amongst tRNA 2 and very rarely in other actors of translation such as rRNA, ribosomal proteins and translation factors that usually act by increasing the ribosomal ambiguity and mistranslation 3,4.

Suppressors located in ribosomal RNA genes are usually very difficult to select due to the redundancy of such genes. A particular suppressor located in the 15S rRNA of *E.coli *has been isolated by 4. On the other hand, the situation is ideal in yeast mitochondria where the exceptional existence of only one copy of the small and large rRNA genes should allow the isolation of rRNA suppressors, if not lethal. Indeed, such suppressors have been isolated already: mim3-1 by 5,6 and MSU1 by 7.

The mim3-1 suppressor is known to act on various ochre mitochondrial mutations and one particular frameshift mutation 5,6,8; see also the text. These mutations are also suppressed genotypically by nuclear informational recessive suppressors, nam3-1 and nam3-2 5,6 and phenotypically by paromomycin 9,10. The MSU1 mutation suppresses only ochre mutations and has occurred at the base of the so called "530-loop" in the 15S rRNA 11. A few years ago, it was shown that the Nam3 protein corresponds to the release factor Mrf1 and the identification of the *nam3-1 *suppressor mutation was also reported 1.

Another nuclear suppressor, Nam9-1, which acts on only few ochre mutations, also suppressed by mim3-1 and nam3-1 suppressors, has been shown to be highly homologous to the S4 ribosomal protein of chloroplasts, bacteria and eukaryotes 12. The mutation responsible for the suppressor phenotype has been identified and shown to be analogous to the *ram* (ribosomal ambiguity) mutation in the *E.coli* S4 gene 13.

We report here the sequence of mim3-1 and show that the mutation has occurred at the same position as in the MSU1 suppressor but has another base replacement. We report also the sequence of various mitochondrial mutations that are suppressed or not suppressed by mim3-1; we propose a mechanism of suppression based on these determinations and mainly on a particular frame-shift suppressed mutation. Furthermore, genetic evidence is given concerning an interesting interaction between the location of mim3-1 suppressor in the mitochondrial 15S rRNA and the product of the nam3-1 nuclear suppressor.

## RESULTS

### Cloning and sequencing of various intronic trans-recessive mutations

The mim3-1 suppressor was shown to be acting on various mitochondrial mutations located in different genes. The majority of them are trans-recessive intronic mutations, few are located in the exonic parts or in uninterrupted genes but none of them is a cis-dominant intronic mutation 5,6. This indicated that these suppressors have most probably touched some components of the translational machinery. We decided to characterize the nature of other target mutations of these two suppressors in order to shed light on the mechanism of suppression and on the possible nature of the suppressors. Besides the already known mutations (see Table 1 and 2), we have chosen four intronic trans-recessive mutations located in the second intron (bi2) of the cytochrome b gene of yeast mitochondria: three are suppressed by mim3-1 and nam3-1 (G5026, G5084 and M2573, Table 1) and one that is not suppressed (G5006, Table 2). They have been already genetically mapped by "deletion cartography" 14,15 using a set of discriminating rho^-^ mutants and by establishing the recombination frequencies between these different mitochondrial mutations (mit-) 8,14. Indeed, we have to recall that all mit- are finely mapped by genetic tools such as the crosses between rho^-^ and mit- as well as between each mit- 8,14,15. In the first set of crosses, several overlapping rho^-^ are used to map the mit- mutations, the rationale is: if the mit- is covered by the rho^-^, it will give respiratory positive diploids, growing on glycerol (N3 plates) whereas both parents (rho^-^ and mit-) didn’t grow on N3 medium. In the second set of crosses, the greater the distance between two mit-, the greater is the percentage of recombination between them.

**Table 1:** Nucleotide sequence of mitochondrial mutations suppressed either by the 15S mitochondrial ribosomal RNA mutation mim3-1 or by the dominant nuclear mutation Nam9-1 encoding the ribosomal protein S4 or by the recessive nuclear mutation nam3-1. The mutated site is given with its surrounding context. The base present in the mutant is indicated in bold characters, above the mutated codon which is underlined. ORF-bi2 and ORF-bi4: Open reading frames coding for RNA-maturase of the second and fourth introns of the cytochrome b gene respectively; COX2: uninterrupted gene encoding the subunit II of cytochrome oxidase; fs: frameshift mutation. The results of suppression are from 5,6 for nam3-1 and mim3-1 and 12 for NAM9-1. The numbers correspond to the distance of the nucleotide sequence, in base pairs, from the first base of the initiation codon ATG.

**Mutant**	**Nucleotide sequence**	**Nature**	**Locus**	**Suppression**			**Reference**
				**mim3-1 ** **nam3-1**	**Paro**	**Nam9**	
**G5026**	** A ** ATT AAT TA**T** TTA TAT Tyr	TAA	bi2+	+	+	2	this work
**G5084**	** A ** AAT ACA T**G**A ACT TAT Trp	TAA	//	+	+	+	//
**M2573**	** A ** GAC AAT TA**T** TTA ACT Tyr	TAA	//	+	+	+	//
**M2075**	** A ** AAC ATT T**T**A TCA ATT Leu	TAA	//	+	+	+	47
**M3041**	I**+A** TTT AGT TAT AAA GAT Tyr	fs	//	+	+	-	43
**M1431**	** A ** AAT GCT T**G**A TTT ATA Trp	TAA	bi4	+	+	-	16
**V25**	** T ** CAT GGA **C**AA ACT ATT Gln	TAA	COXII	+	+	-	7

**Table 2:** Nucleotide Nucleotide sequence of mitochondrial mutations suppressed neither by mim3-1, NAM9-1 nor by nam3-1.ORF-bi2 and ORF-bi3: Open reading frames of the introns bi2 and bi3; B1 and B3: first and third exon of the cytochrome b gene. fs: frameshift mutation; ms: missense mutation.

**Mutant**	**Nucleotide sequence**	**Nature**	**Locus**	**Suppression**			**Reference**
				**mim3-1 ** **nam3-1**	**Paro**	**Nam9**	
**G5006**	** I+A ** AAA GAT GTT CAA TAT Val	fs	bi2	-	-	-	this work
**G171**	** T ** TAT GGA **C**AG ATG TCA Gln	TAG	B1	-	-	-	43
**M6821**	** A ** GGT TTA TA**T** TAT GGT Tyr	TAA	//	-	-	-	48
**W91**	**A** CCT ATT T**T**A ACT AAA Leu	TAA	bi2	-	-	-	43
**M7793**	**-A ** AGA AGA AAA TTA GCA Lys	fs	bi3	-	-	-	41
**G5037**	**A** TTA ATT G**G**T TTT TTT Gly	ms	//	-	-	-	49
**M2011**	**G** AAT TTA T**T**C TCA GCA Phe	ms	B3	-	-	-	49

Once these four mutations were cloned and sequenced as described in Materials and Methods, the physical and genetic map showed a great concordance 8. All mutations were simple and the predicted length of the mutated proteins was in full agreement with the molecular weight determined by SDS-PAGE 16.

In the case of the G5084 mutant, besides the first intronic mutation, a second missense mutation was discovered, which abolishes a BglII restriction site in the beginning of the last exon B6 in the cytochrome b gene; AGATCT becomes AGATAT. Although this second mutation converts a Serine to a Tyrosine, this change is silent since the diploid obtained by crossing the G5084 mutant to the B231 petite, which covers only the first change of G5084, grows well on N3 medium (rich medium containing glycerol), i.e the recombination between B231 rho^-^ and G5084 gave diploids that are respiratory competent since they grow on glycerol. We remind that glycerol is a respiration substrate, not suitable for fermentation, while glucose can be either fermented or respired. This result is unexpected since the position and the nature of the G5084 second change seems to be structurally important for cytochrome b (J.P di Rago, personal communication). We have also to keep in mind that all the mutants sequenced in the current work were derived from the 777-3A wild type strain, that don’t have the second change found in G5084. Whatever the explanation, the first change of G5084 (Table 1) is responsible of its respiratory deficiency and is therefore the target of both mim3-1 and nam3-1 suppressors.

**Figure 1:**
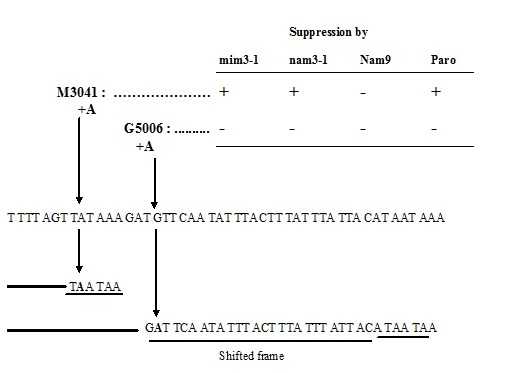
FIGURE 1: Nucleotide sequence of suppressed and non-suppressed frameshift mutations in the RNA-maturase coding part of the second intron of the cytochrome b gene. Note that both mutations create double ochre stop codons, one immediately after the addition of adenine (M3041) and the other after a shift of nine amino acids after the addition of adenine (G5006). Numbering like in table 1.

The sequences we determined were compiled in Table 1 and 2, as were all of the known mutations on which the action of mim3-1 and nam3-1 suppressors was tested. Six suppressed mutations are shown to be mono-substitutions, creating ochre (UAA) stop codons, and only one, M3041, is a frame-shift. This latter mutation created in addition two ochre stop codons at the site where the A insertion occurred (see Figure 1).

Among the non-suppressed mutations, we found all different types of mutations: nonsense (three ochres and one amber), two frame-shifts (one mono-addition and one mono-substraction) and finally two missense mutations (Table 2). Interestingly, the non-suppressed mono-addition, G5006, has taken place very close to M3041 (Figure 1). From these results we can conclude that, although there is a relevant clear bias towards ochre stop codons, the mim3-1 and nam3-1 ribosomes act preferentially on ochre stop codons and only one particular frame-shift mutation. The suppression of the M3041 frame-shift mutation is very intriguing and will be discussed further in detail (see Discussion).

### Genetic and physical mapping of the mim3-1 suppressor using "rho^-^ cartography"

Ethidium bromide mutagenesis is usually used to generate rho^-^ (petites, i.e cells with a mitochondira retaining a fragment of its mitochondrial DNA) and rho^0^ (cells with mitochondria devoid of mitochondrial DNA) at a very high rate (at % scale) from any given strain 14,15. If the starter strain contains a mitochondrial mutation, some rho^-^ would contain such mutation in the retained mitochondrial DNA and could be thus used to map the mutation. Using such technology, the mim3-1 suppressor mutation was localised in a rho^- ^covering the 15S rRNA region, between the COX1 and COX3 genes 5.

**Figure 2:**
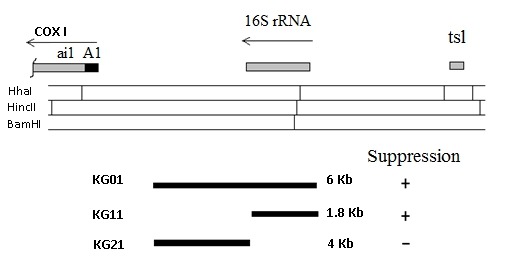
FIGURE 2: Physical mapping of the mim3-1 suppressor. The restriction map and position of 15s rRNA and COX1 (subunit I of cytochrome oxidase) genes are shown at the top. tsl: locus involved in tRNA maturation. The KG01 rho^-^ petite, carrying the mim3-1 mutation, was subjected to ethidium bromide mutagenesis and sub-cloned twice in the absence of the drug. A few hundred colonies were tested for suppression of the M3041 target mutation. + and - indicates suppression or its absence. Most of the sub-clones did not suppress any longer; 24 rho^-^ sub-clones were further analysed by restriction (only a few sites are shown) and blotting with a probe constituted by the KG01 mitochondrial DNA. The lengths and positions of tandem repeats retained in mtDNA of three discriminating petites are represented by thick bars indicating that the suppressor is located in the 15s rRNA gene.

We have submitted this rho^-^ strain, that we call here KG01, to ethidium bromide mutagenesis 14,15 in order to select petites shorter in length. From 24 such petites, analysed by restriction mapping, two novel petites were selected, one retaining the suppressor ability (KG11) and one having lost it (KG21). The mitochondrial DNA was extracted from these petites and submitted to restriction mapping and southern hybridization using the KG01 mtDNA as a probe. The restriction enzyme HhaI was chosen as to allow us to know the length of the 15S rRNA gene. Conclusions are summarized in Figure 2. The KG11 petite suppressed the M3041 target mutation; its mtDNA hybridized to the probe and appeared to contain the entire 15s rRNA gene. KG11, 1.8 Kbp long, was completely sequenced.

### Nucleotide sequence of the mitochondrial 15s rRNA gene of the mim3-1 suppressor and comparison with wild-type 15S rRNA sequences

**Figure 3:**
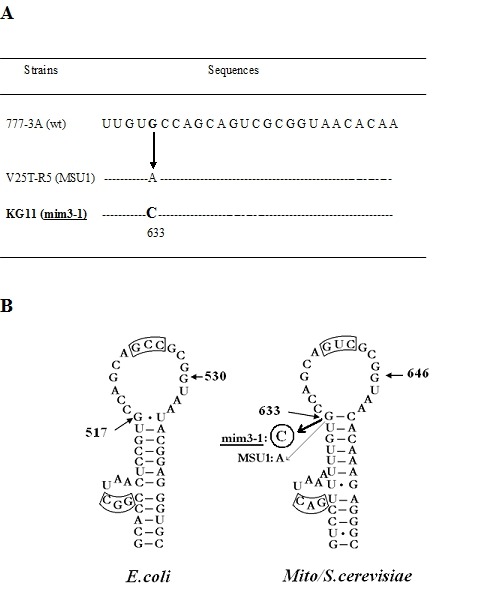
FIGURE 3: **(A) ** Nucleotide sequence of the mim3-1 region in the 15S rRNA. The nucleotide sequence of 629-754 region in the mitochondrial 15S rRNA of various strains is shown. Sequences are from: 1-50; 2: 51; 3: 17; 4 and 5: 11. The position 633 is the only one mutated in the ochre suppressors MSU1 and mim3-1. The underlined bases are engaged in a stem structure. **(B) ** Position of mim3-1 on the 530 stem-loop secondary structure. On the right the structure of yeast mitochondrial 15s rRNA is shown which is homologous to the 530 bacterial one, numbered from 5'. The G633 which is replaced by C in mim3-1 and by A in MSU1 suppressors 11 is boxed. On the left the 530 stem-loop structure of the 15S rRNA in *Eubacteria* is shown with numbering according to *E.coli*. The substitution of G530 (circled) by either C, A or U is lethal in *E.coli* 28. Note that the boxed triplet in both structures is complementary and may interact in the tertiary structure according to 22,23.

The complete nucleotide sequence of the mitochondrial 15S rRNA gene and surrounding 5' and 3' regions was then established using primers synthesized according to the known sequence of wild type 15S mt-rRNA. The nucleotide sequence of mim3-1 15S rRNA gene was compared to the wild type sequence of the isogenic strain 777-3A 11,17. Only one difference was noted: the G633 (corresponding to G517 in the *E.coli *16S rRNA) is changed to C, Figure 3A. This change has touched a highly conserved base in several hundreds of 15S rRNA mitochondrial sequences. This base is known to interact with a C or U in the rRNA secondary structure and to be at the junction between an invariant stem and a loop, as shown in Figure 3B. We have to note that polymorphisms between the 15S rRNA genes of various yeast strains have been previously reported 17,18. The 15S sequence for the strain KG11 is completely identical to that reported for the 777-3A wild-type strain while the MSUI strain 11 contained the bases 5'-AGATTAAGTT-3' at position 1136-1145 instead of 5'-AGATAATGTT-3' (777-3A) 11,17,18.

### Interaction between nam3-1 and mim3-1 suppressors

The nam3-1 suppressor, isolated by 5, acts roughly on the same mitochondrial mutations as mim3-1. This suppressor is nuclear and, due to its informational character and to the fact that all of the mitoribosomal proteins are nuclearly encoded, before the publication of the work of 1 it was believed that this mutation probably lies in a gene encoding a mitoribosomal protein or a component of the mitochondrial translational machinery. This hypothesis encouraged us to answer the next question: is there any interaction (synergism, antagonism or neutrality) between the mim3-1 and nam3-1 suppressors?

A strain bearing the nam3-1 suppressor and devoid of mtDNA (so qualified as rho^0^) was crossed with a strain bearing in its nucleus the Nam3+ wild type allele and in its mitochondria the mim3-1 suppressor mutation and a suppressible target mutation, M3041, i.e [mim3-1, M3041] mitochondrial genotype. Since the first strain carried also the *kar1-1 *nuclear mutation (characterized by a retardation in karyogamy 19) the introduction by cytoduction of the [mim3-1, M3041] mitochondria into the rho^0^ nam3-1 cell was possible. Such genetic tool is called “cytoduction” in yeast mitochondrial genetics 19.

**Figure 4:**
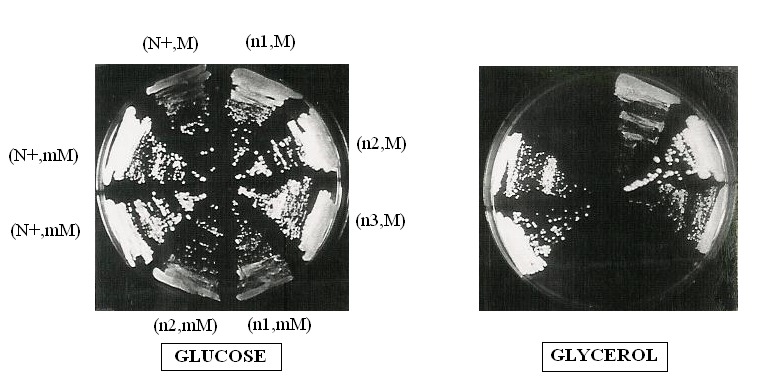
FIGURE 4: Haploid cells carrying both mim3-1 and nam3-1 suppressors are respiratory deficient. Fresh cultures of different strains are streaked on YPGA (glucose) and N3 (glycerol) plates and incubated three days at 28°C. All of these strains are isogenic: the nucleus is from JC8 and the mitochondria are from 777-3A. The nuclear genotype is given in brackets (with the nuclear one at the left and the mitochondrial at the right) with N+: NAM3^+^, n1: nam3-1; n2: nam3-2, n3: nam3-3; m: mim3-1; M: M3041.

All the triple mutant cytoductants [nam3-1, mim3-1, M3041] were shown to be completely respiratory negative (Figure 4). On the other hand, all the diploids were respiratory competent (data nor shown). We should note that in cytoduction experiments, not only cytoductants are obtained but also diploids because the kar1-1 mutation didn’t abolish karyogamy but retardates it 19. These diploids [Nam3+/nam3-1, mim3-1, M3041] are respiratory competent because, as nam3-1 is recessive, it cannot disturb the action of mim3-1, the suppression of M3041 is therefore fulfilled by mim3-1 alone. The cytoductants were identified nuclearly by their auxotrophic markers and mitochondrially by crossing with the FR111 petite (which covers the M3041 mutation) and with the F0 rho^0^ strain (FR111 and F0 are isonuclear). These two petites restored the respiration to the majority of the triple mutants, see below.

Moreover and interestingly, each of the triple mutant cytoductants was rapidly spontaneously converted into a population of petites after culture on glucose rich medium. Thus, the presence of the two suppressors in the same haploid cell not only hampered respiration but led to the loss of mitochondrial DNA integrity, due to complete disorganisation of mitochondrial translation. The effect of each suppressor alone on "petite formation frequency" was tested (Table 3). In an isonuclear background, nam3-1 seems to be more deleterious than the mim3-1 suppressor. Consequently, due to this high rate of “petite” formation nam3-1 and nam3-2 colonies presented an irregular contour on glucose medium whereas mim3-1 colonies exhibited a normal shape (I versus N, Table 3). The same table shows also that the nuclear context modulates the effect of nam3-1 mutation on the petite formation frequency (compare the effect of nam3-1 in the two nuclear backgrounds 1 and 2, Figure 4 and Table 3).

**Table 3:** Frequency of cytoplasmic petite formation in mim3-1 and nam3-1 suppressor strains. All strains are isogenic, contain the nucleus of JC8, the mitochondrion of 777-3A/M3041 and differ only by the nuclear mutation nam3 or the mitochondrial mutation mim3. The method is outlined in Materials and Methods. The appearance of the colonies on YPGA solid medium is given: irregular and normal shape.

**Genotype**		**Pt**	**P0**	**N**	**N0**	**n**	**Mutation**	**Appearance**
**Nucleus**	**Mitoch.**			**(x10^8^)**	**(x10^8^)**		**rate**	
nam3-1 (1)	M3041	70	5	108	250	25.Jul	0.044	Irregular
nam3-2 (1)	M3041	66	3	180	240	26.Apr	0.040	Irregular
Nam3+ (1)	mim3-1, M3041	18	0.8	55	300	24.Feb	0.007	Normal

## DISCUSSION

We have identified the mim3-1 suppressor as a substitution of G633 by C in yeast mitochondrial 15S rRNA. The most interesting finding, in this context, is that this base is also substituted by A in another mitochondrial suppressor, MSU1 11. This G, corresponding to the G517 in the *E.coli *16S rRNA sequence, was also mutated in *E.coli *and shown to become able to suppress either nonsense as well as frameshift mutations, demonstrating the role of G517 in translation ambiguity 20. G517 is invariant among more than 100 known 15S rRNA sequences 18 and argues for an important role in translation. Its position in the secondary structure is also invariant and important since it is at the top of a highly conserved stem (Figure 3B). Even at the level of tertiary structure, this region is very important since a functional pseudoknot was reported there 21,22. Indeed, the potential base pairing between residues 524-526 in the 530 loop and the 510 bulged region is highly conserved between bacteria, archebacteria and mitochondria 23; see on Figure 3B the boxed triplets. Such peudoknots seem to be unique and situated in the most conserved catalytic region of highly structured RNA, such as RNase P RNA 24 and group I self-splicing introns 25, a fact that underlines their functionality 22. It is highly likely that such higher-order structural interaction should be affected by mim3-1 or MSU1 substitutions since the structural basis of the loop (the G-C base pair) is destroyed. A more opened 530 loop would be in favour of an enhanced read-through, as suggested by 26.

This so called 530 stem-loop in *E.coli *is very important for many reasons. The 530 stem-loop interacts with three ribosomal proteins: S4, S5 and S12. The pseudo knot is covered and stabilized by the S12 protein, which protects it against ketohexal and dimethylsulfoxide 22. These authors consider S12 as the most important ribosomal protein at the functional level. It should be noted that - *in vivo* - the ribosomal proteins S12, S4 and S5 were involved in nonsense read-through in *Bacillus subtilis *27. It was shown that the substitution of G530 by A, C or U result in dominant lethal mutations in *E.coli *28. The “G530 to A” context is lethal since the functionality of EF-Tu is hardly affected, leading Powers and Noller to conclude that the ribosome modulates this activity during translation by "affecting the speed and/or the accuracy of tRNA selection" 29.

The fact that the majority of the suppressed mutations are leaky 8,30 suggest that mim3-1 and nam3-1 suppressors, as well as paromomycin, didn't create a new situation but "accentuate" a pre-existing one, for instance the ribosome ambiguity and/or slippage. This could result from the action of a modified ribosome or due to the modification of an elongation or even a release factor. It is known that certain mutations in the EF-Tu elongation factor suppress nonsense and frameshift mutations in bacteria 31,32 and in yeast 33. In this context, it should be noted that the location of interaction between EF-Tu and the large ribosome subunit is adjacent to the "530" loop 34.

The decoding site (position 1400-1500 in the *E.coli *16S rRNA) and the 530 loop are located side by side in the 30s subunit. The protection by tRNA of the 530 region is in fact an allosteric result of the tRNA interaction at the decoding site and involves S1, S3 and S5 proteins 29,35, but the S12 protein is also in functional relation with the 2660 region of the 23s rRNA, "the alpha Sarcin loop" region 36,37. Therefore, since the 530 loop is adjacent to the fixation site of EF-Tu and G factors, this would involve this loop in some factor-mediated communication with the 50S subunit. In this context, it was shown that a G to C mutation at position 2660 hampers elongation but, surprisingly, only in the presence of StreptomycinR mutations in the S12 protein, is this functional impairment suppressed by a third mutation in EF-Tu itself 38. It was also suggested that the ribosome frameshifting is dependent on the concentration of mutated EF-Tu 39. This could explain the recessivity of a given suppressor mutation.

The data advanced above suggest a direct participation of the 530 stem-loop in ribosomal function, particularly in the control of translational fidelity and ribosomal ambiguity, and led us to propose that the nam3-1 encoded protein should be a constituent of ribosomes or it must interact with mitochondrial ribosomal proteins (the counterparts of the *E.coli *S4, S5 or S12 proteins). Indeed, in this work, we showed that nam3-1 antagonizes the effect of the mim3-1 suppressor. When both suppressors are present in the same haploid cell, the suppression of a target mutation ceases and the mitochondrial DNA degenerates rapidly into "petites". In fact, we may suspect that translation becomes excessively erroneous and/or perhaps that mitoribosome assembly would not be possible. Consequently, the mtDNA integrity is abolished (see Table 3 and 4).

**Table 4:** Summary of interaction between mim3-1 and nam3-1 ochre suppressors and their effects on mitochondria. The table summarizes the individual effects of each suppressor on the mitochondrial ribosome assembly, translation and DNA integrity. When both suppressors are present, all the cells become rho^-^/rho^0^, see text for more details.

**Genotype**		**Mitochondrial function status**		
**Nucleus**	**Mitoch.**	**Ribosome**	**Protein synthesis**	**DNA integrity**
NAM3+	mim3+	Normal	Present (TAA is stop)	Normal
NAM3+	mim3-1	Increased ambiguity	Present (TAA is read)	Slightly Decreased
nam3-1	mim3+	Increased ambiguity	Present (TAA is read)	Decreased
nam3-1	mim3-1	Excessive ambiguity or not assembled	Completely erroneous or absent	Abolished

Another view of this interaction between both suppressors would claim that combination of mim3-1 and nam3-1 could result in a kind of “super-suppressor” due to the synergistic action of the two suppressors, which no longer allows correct termination and reading frame maintenance. In similar context, Cox claimed that “ochre suppression itself is potentially lethal when it becomes too efficient” 40.

Whatever the explanation, the functional interaction between mim3-1 and nam3-1 mutated products is concordant with the fact that the Nam3 gene encoded a mitoribosome constituent: the release factor (mRF1) 1. The group of Magdalena Boguta disclosed also the nature of the nam3-1 and nam3-2 suppressor mutations, called respectively *mrf1-145 and mrf1-136, *at residues (S352I) and (S216Y) 1. These mutations were found in the highly conserved «domain 2» which is supposed to be responsible for stop codon recognition and contains the motif PST. This motif is believed by these authors to bind directly to stop codons in mitochondrial mRNA and contacts helix 44 of 15S rRNA 1. Since Helix 44 is involved in the interaction between the ribosomal subunits, we understand the importance of this interaction on the ribosome functionality. It is worthy to note that these authors described the effect of two mutations (Q349R and S352I (nam3-1)) on the physicochemical properties of the surface of domain 2, which might affect its direct binding to the ribosome (ribosomal protein S12 and 15S rRNA), strengthening therefore our previous conclusions.

We have shown that the diploids (Nam3+/nam3-1) bearing a [mim3-1, M3041] mitochondria are respiratory competent cells. Thus, a single copy of the Nam3 wild type gene is sufficient to alleviate the negative interaction between mim3-1 and nam3-1 (or nam3-2), in accord with the already known recessivity of the nam3-1 and nam3-2 mutations 5. This recessivity is to opposite to the dominant lethal substitutions of G530 in the 16S rRNA of *E. coli *28. In fact, it should be interesting to substitute, in the *E.coli *16S rRNA gene, G517 to C, A and G in order to test their suppression capacity, if they are not lethal.

As both mim3-1 and MSU1 suppressors act preferentially on ochre mutations, it is highly likely that the 530 stem-loop plays an important function in the read-through of UAA codons. Interestingly, it should be noted that firstly the mim3-1 ribosome suppresses efficiently one particular frameshift mutation, M3041. This mutation creates two ochre stop codons exactly where the addition has occurred, without any frameshifting. M3041 could be assimilated in this way to a simple nonsense substitution since it didn’t create a frame shifting. It is worth noting that another +A frameshift mutation in intron bi2, G5006, which is very close to M3041 mutation and which creates also two ochre stop codons after a few shifted codons, is not suppressed by mim3-1, nam3-1 nor by paromomycin (Figure 1, Table 2).

From all the data (Table 1 and 2), we propose that the mito-ribosome would have an enhanced capacity of slippage, just when stalled at a mutated ochre codon (the majority of the classical nonsense mutations and the particular (+1) M3041 mutation). We shall note here that Towpik *et al. *1 suggested that some particular amino acid substitutions in mutated mRF1 would lead to ribosomal stalling. In addition, such ochre codons should be in a “favourable context". Such codon context effect has been shown already in some tRNA suppressors, mainly in bacteria 41. A favorable context could be considered in two different ways: (1) the mim3-ribosome can slip only in certain region of the mRNA and only mutations situated in these regions are suppressed; (2) the mim3-1-ribosome can slip everywhere but only certain regions of the translated protein can tolerate the insertion of small stretches of false amino acids; therefore the mutations situated in the corresponding regions on DNA are suppressed.

## MATERIALS AND METHODS

### Strains

The rho^-^ strain bearing the mim3-1 mutation, CK247/B291/73, was isolated by 5, it is named here KG01. The rho^0^ strains bearing the nam3-1 suppressor, CK311/B145/50, and the nam3-2 allele, CK311/136/50 were also constructed by 5. The mit- 777-3A/M3041 mutation was already sequenced, it is a frame-shift mutation in the maturase part of the second intron bi2 16. This mutation can revert to respiration competence by classical suppression, such as by mim3-1 and nam3-1, or by intron deletion 30,42. FR111 is a rho^-^ deriving from D273-10B, lacking the three first introns bi1, bi2 and bi3 of the cytochrome b gene and extending from the beginning of the cytochrome b gene to the beginning of the bi4 intron 43.

### Media

The solid media used are: YPGA (rich medium, non-selective for respiration): 1% yeast extract, 1% bacto-peptone, 2% glucose, 2% agar and 60 mg/l adenine; N3 (rich medium, selective for respiration): 1% yeast extract, 1% bacto-peptone, 2% glycerol and 2% agar, adjusted to pH6 with 50mM phosphate buffer; YPD, rich medium, differential medium (the respiratory positive cells grow better than the negative ones): 1% yeast extract, 1% bacto-peptone, 2% glycerol, 2% agar and 0.1% glucose; W0 (minimal medium, selective for diploids): 0.67% Yeast Nitrogen Base, 2% glucose and 2% agar; W0FL (minimal medium, selective for cytoductants of [a] genotype): as W0 with 20mg/l leucine and 20 mg/l canavanine. The liquid media are YPGA, as the solid medium without Agar, and YP10 as YPGA but with 10% glucose.

### EtBr mutagenesis, crosses and cytoduction

These different genetic techniques were conducted as in 9.

### Determination of the frequency of cytoplasmic petite formation

The tested strains are respiratory competent and were first grown on a glycerol containing medium (N3) and the proportion of respiratory deficient cells in the culture was determined by replica plating on N3 and YPGA (containing glucose) solid medium, giving the value of P0. Then the cultures were appropriately diluted and inoculated into a high glucose containing medium (YP10) for the indicated number of generations (n = Ln(N) - Ln(N0) / Ln2), with N0 and N as the number of cells at time zero and at the end of the culture. The proportion of respiratory deficient cells was determined again by replica plating on N3 and YPGA, giving the value of Pt. The mutation rate (m.rate) rho^+^ to rho^-^ is given by 1/n x Ln ((1-P0) / (1-Pt)) 44.

### Extraction of mtDNA and plasmid DNA

The mitochondrial DNA was extracted according to 45. The plasmid and phage DNA was extracted from *E.coli *according to 46 with minor modifications.

### Cloning and sequencing

The shotgun cloning of mitochondrial mutations was realized in pBR322 vector as follows: the mitochondrial DNA digested by BamHI and BglII is ligated to the BamHI digested vector. The recombinant plasmids containing the 1.5 kbp fragment, encompassing the entire bi2 intron and the surrounding exons, were selected using a bi2 specific probe. The cloning of the mitochondrial 15S rRNA gene in the replicative forms mp19 and mp18 of the phage M13 was achieved in order to determine the entire sequence of the mim3-1 mutated gene. Oligonucleotides were synthesized, according to the wild type 15S rRNA mitochondrial sequence 17.
